# Manchester Infirmary

**Published:** 1830-02

**Authors:** 


### MANCHESTER INFIRMARY.

### *Case of Paralysis cured by Strychnia.*

JOHN PRINCE, twenty-nine years of age, spinner, admitted an in-patient September 13th, 1824, was seized, about six months ago, with loss of power in the lower extremities, after bathing, whilst the body was much heated with exercise. He is now incapable of motion without the aid of crutches. He passes his urine and feces involuntarily. The spine is free from pain. His strength is much reduced; appetite bad; pulse seventy-two, and rather feeble. I directed him to take Pil. Hydrargyri four grains each night, with a saline aperient on the following morning, for the first ten days. On the 24th, I commenced with the strychnia, in the dose of a sixth of a grain, three times daily.

October 4th.—The alkali has not produced any effect upon him. Appetite somewhat improved; in other respects he remains in the same state as on his admission.

10th.—Strychnia augmented to the fourth of a grain every fourth hour.

14th.—Has experienced severe convulsive twitchings in the affected limbs. He is sensible of an increase of power in his inferior extremities, and wishes to rise and make trial of his crutches.

22d.—Is very much better. Strychnia to be taken in the proportion of half a grain three times daily.

November 4th.—During this interval the alkali has been attended with great benefit. He is now capable of retaining both urine and feces, and of walking from one end of the ward to the other with the aid of a small stick. His appetite is good, bowels regular, and spirits cheerful. To continue the alkali.

16th.—He is entirely cured. In order to show the pupils of the hospital what he could do, he ran from one end of the long gallery to the other. I ordered him to be discharged at the first meeting of the weekly board.

I had an opportunity of seeing this patient several times after he left the house, and was glad to find that he continued to enjoy the perfect use of his lower limbs.—*Bardsley's Hospital Facts.*

---

### *Cases of Neuralgia, in which the Acetate of Morphia was successfully employed.*

I. Ann Jones, forty-five years of age, unmarried, had enjoyed a tolerably good state of health previously to last April (1826), when she was first seized with an acute pain, shooting from the fore part of the left cheek to the ala of the nose and angle of the mouth of the same side. Since that time she experienced a return of the pain, at least once each week, until within the last two months, when it has recurred several times daily. Her general health is much impaired by the constant irritation of the complaint, and her flesh and strength are greatly reduced. She has been in the habit of taking ether in large doses, from which she has mostly derived a temporary mitigation of her agony. Leeches, blisters, moxa behind the ear, opiate liniments, friction with the lead ointment (as recommended by Mr. Bedingfield,) were first employed externally, and subsequently purgatives, antispasmodics, tonics of various kinds, and carbonate of iron internally, but with little or no benefit.

Under these circumstances I commenced with the acetate of morphia, in the dose of a quarter of a grain every second hour. In the course of two days the proportion was increased to half a grain at the same intervals. On the third day, the beneficial effects of the remedy were duly appreciated by the patient. She persevered with the dose of half a grain, twice daily, for more than three weeks, at the expiration of which time the disease had completely yielded to the influence of the morphia. She has remained well ever since.

I only found it necessary to order her opening medicine twice during the above period.

II. Mary Williamson, fifty-two years of age, admitted a home-patient, June 6th, 1825.

She was attacked, about three months ago, with a severe lancinating pain in the situation of the right mental foramen, which continued for several hours, and then ceased. It returned, however, on the next morning with aggravated violence, and left her again in the evening. As the pain extended to the teeth on the same side of the jaw, she had been persuaded to have two of them extracted, but the operation afforded her no relief. The remaining teeth appeared to be perfectly sound. When she came under my care, the least degree of motion, or any attempt at mastication, caused a return of pain. Her general health was much impaired, from the constant irritation to which she was exposed, and she had lost both flesh and strength. Feeling satisfied, from the above symptoms, that the inferior dental nerve was affected, I resolved upon the employment of the acetate of morphia. She was accordingly directed to commence with a quarter of a grain every fourth hour, after the bowels had been freely opened by purgative medicine.

June 20th.—She has had no pain during the last seven days, and can now eat and take exercise without fear of its recurrence. Appetite better; bowels regular. Half a grain of the salt to be taken once daily.

July 12th.—She is in her usual state of health. Ordered to be discharged cured.

I saw this woman several times afterwards, and was glad to find that the pain had not once returned.—*Ibid.*

